# What Are the Causes of Death among Patients Admitted to a Contemporary Tertiary-Level Cardiology Department? An Analysis of 10 Years of Morbidity and Mortality Meetings

**DOI:** 10.3390/pathophysiology30040034

**Published:** 2023-09-30

**Authors:** Chun Shing Kwok, Jacopo Tafuro, Chun Wai Wong, Sadie Bennett, Donah Zachariah, Diane Barker, Adrian Morley-Davies, Duwarakan Satchithananda, Mark Gunning, Josip A. Borovac

**Affiliations:** 1 Department of Cardiology, University Hospitals of North Midlands NHS Trust, Stoke-on-Trent ST4 6QG, UK; shingkwok@doctors.org.uk (C.S.K.); jacopo.tafuro@uhnm.nhs.uk (J.T.); wongcw@doctors.org.uk (C.W.W.); donahez@me.com (D.Z.); diane.barker@uhnm.nhs.uk (D.B.); adrian.morley-davies@uhnm.nhs.uk (A.M.-D.); duwarakan.satchithananda@uhnm.nhs.uk (D.S.); mark.gunning@uhnm.nhs.uk (M.G.); 2 Faculty of Health, Education and Life Sciences, Birmingham City University, Birmingham B5 5JU, UK; 3 Division of Interventional Cardiology, Cardiovascular Diseases Department, University Hospital of Split, 21000 Split, Croatia

**Keywords:** death, acute myocardial infarction, heart failure, cardiac arrest, cardiovascular departments, sepsis, causes of death, outcomes, medical ward

## Abstract

Despite the efforts to deliver the best evidence-based care, in-hospital death is an inevitable event among some patients hospitalized in cardiology departments. We conducted a retrospective evaluation of mortality events from inpatient admissions to the cardiology department between 2010 and 2019. Data were collected from morbidity and mortality meeting presentations that evaluated comorbidities, medical history, treatments, and causes of death for the overall cohort and according to age group and sex. There were 1182 registered deaths. The most common causes of death among patients were acute myocardial infarction (AMI, 53.0%), heart failure (HF, 11.7%), cardiac arrest (CA, 6.6%), HF with complication/defined cardiomyopathy (6.3%), and sepsis (4.4%). We observed a decline in deaths from AMI from 61.9% in 2010 to 46.7% in 2019, while there was a clear increase in deaths from HF (11.1% in 2010 to 25.9% in 2019). Compared to patients ≥65 years, younger patients were more likely to have died from CA (15.7% vs. 4.3%, *p* < 0.001) and other cardiac reasons (3.0% vs. 0.4%, *p* < 0.001). The majority of deaths were due to AMI, HF, and CA. We observed a significant declining trend in the proportion of deaths due to AMI in recent years, with an increase in deaths due to HF.

## 1. Introduction

Despite decades of research, medical advances, and evidence-based care, general inpatient hospital mortality remains around 2% and is an integral part of everyday clinical practice. In cardiology departments, healthcare professionals manage patients who are often acutely ill and may deteriorate suddenly. An important facet of the continued effort to improve clinical practice and maintain high standards of care is the evaluation of performance and reflection on how clinical activities impact patient outcomes. At the cardiology department of our institution, a monthly morbidity and mortality (M&M) meeting is held regularly, where all deaths are discussed, and the National Confidential Enquiry into Patient Outcome and Death (NCEPOD) classification of good clinical practice is documented [1]. M&M meetings play an important role in improving the accountability of mortality data, the quality of patient care, and safety and may foster inter-professional learning among providers [2,3,4,5].

The M&M meetings have an educational role for physicians and other healthcare providers and can help in preventing medicolegal issues [6]. In the process of reviewing all deaths, less common conditions will occasionally be brought to the forefront. This is important for doctors and nurses in training, as it helps to broaden their awareness of the clinical spectrum as a whole [7]. Furthermore, a review of patient characteristics can help recognize which patient population is at greatest risk of death. This may inform the development of clinical protocols. In addition, the identification of patterns of the causes of death may highlight areas of care that can benefit from improvements to the service. It should be acknowledged that not all deaths within a cardiology department will be cardiac in etiology. Improved triaging of patients can identify those who may be better managed elsewhere. Of note, non-cardiovascular death is responsible for up to 30% of deaths in patients hospitalized in cardiology wards with acute heart failure [8], while Vicent and colleagues similarly reported that nearly 27% of patients dying in a cardiology department during a 5-year period had non-cardiac causes of death [9]. It is also relevant to ascertain the length of stay and its relationship to the cause of death. For example, the duration of hospital admission was reported to be shorter among patients who died because of cardiovascular causes than among those with non-cardiovascular causes of death [10].

In this work, we collated and analyzed the mortality records from a 10-year span of regular monthly M&M meetings in a tertiary-level cardiology department. To our knowledge, analysis of this kind has not been reported thus far in the medical literature.

## 2. Materials and Methods

The Royal Stoke University Hospital is a tertiary-level hospital providing more than 1300 beds, while the cardiology department delivers a 24/7 primary percutaneous coronary intervention service, structural interventions, device implantations, electrophysiology, heart failure care, cardiac imaging, and outpatient cardiology service. The unit serves a population of approximately 1.4 million patients, and 600,000 are from associated district general hospitals. The monthly M&M meeting incorporates all patients who have succumbed over the preceding month under the auspices of the department. The presentations include information about the age, sex, comorbidities, duration of hospital stay, clinical events, and NCEPOD classification of care received by the patient, as well as the cause of death [11]. As a part of a clinical audit and health service evaluation, we collated data from the morbidity and mortality meeting presentations from 2010 to 2019, incorporating all of the parameters described above. Additional information was gleaned from the hospital’s electronic patient records.

The comprehensive evaluation of the causes of death took place in a cardiology department that has more than one dozen consultants who oversee patient care, and the M&M meetings are important sessions where all the consultants meet to review the deaths that took place within the department the prior month. This is an opportunity for clinical practice to be reviewed, and both good care and care that requires improvement can be identified. Each patient who died had one or more PowerPoint slides describing what happened to them while they were in the hospital. The presented data were based on a detailed review of clinical records in a process that includes a verification of the likely cause of death. In some cases, the cause of death was not certain and was discussed with a coroner. The cause of death was, therefore, based on the clinical records, the consultant’s judgment on the case, and, in some cases, the report of the coroner. The NCEPOD description was determined in the discussion among consultants and junior doctors after the case was presented.

The cause of death was categorized on three levels. The lowest level of cause of death was based on the description in the mortality meeting record or clinical history when the case notes were unavailable. The next level grouped the diagnosis into one of several categories, including acute myocardial infarction (AMI), AMI with non-heart failure complication, aortic stenosis (AS), AS with complication, aortic pathology, arrhythmia, isolated cardiac arrest, cardiac tamponade, heart failure (in a specific sense—defined as an end-stage progression of frank pump failure without any other obvious complications or concomitant conditions significantly complicating the course of the disease), heart failure with complication (e.g., sepsis, complete heart block, bleeding, etc.) or a specific cause (e.g., defined cardiomyopathy such as dilated cardiomyopathy), infective endocarditis, malignancy, other cardiac condition, other non-cardiac condition, pulmonary embolism, sepsis, and stroke. The highest level then regrouped all deaths into the following: all AMI, all HF, isolated cardiac arrest, other cardiac condition, and other non-cardiac condition. In order to limit bias, we initially classified the cause of death between three independent reviewers, and the subgroup classifications were validated independently by a consultant.

Statistical analysis was performed in Microsoft Excel and Stata 14.0 (College Station, TX, USA). The proportion of each cause of death on the highest level was presented in a pie chart. The trends in the proportion of individual causes of death were also evaluated. The median length of stay and number of comorbidities, along with the interquartile range, are shown in a bar chart. A table was used to show the proportion of causes of death overall, according to age <65 years or age ≥65 years, and whether the patient was male or female. In addition, another table was used to describe the causes of death at the lowest level for each subgroup for cause of death. Chi-square tests were used to determine differences in causes of death between older and younger patients, as well as between males and females. In all instances, *p*-values of <0.05 were considered to be statistically significant.

## 3. Results

**Figure 1:**
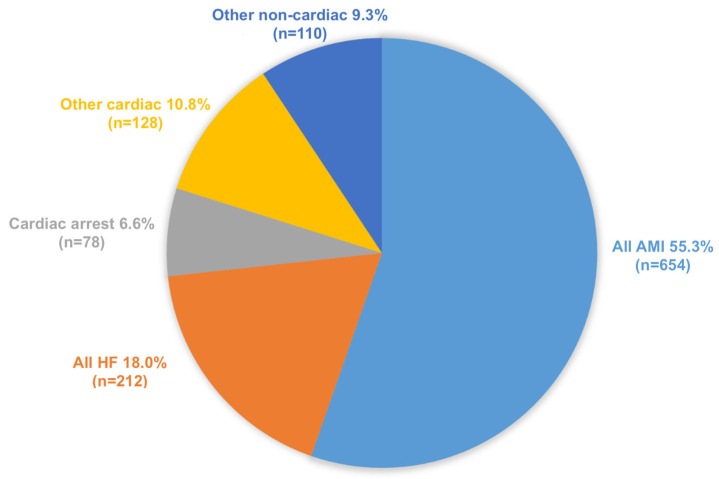
Proportion of major causes of death among patients hospitalized in cardiology department during a 10-year period (from 2010 to 2019).

Between 2010 and 2019, 1182 deaths were captured in our mortality and morbidity meetings. The average age of death was 75 ± 25 years, and 63% of patients were male. More than half of the deaths were due to acute myocardial infarction (55.3%), while heart failure (18.0%), isolated cardiac arrest (6.6%), and other causes made up the remaining causes of death (Figure 1).

Table 1 shows the trends in the proportions of various death causes over time. Comparing 2010 to 2019, there was a decrease in the proportion of deaths due to AMI (61.9% in 2010, 46.7% in 2019) and an increase in the proportion of deaths due to heart failure (11.1% in 2010, 25.9% in 2019). Cardiac causes of death made up the majority, and this proportion increased from 77.8% in 2010 to 88.1% in 2019. The median patient age at the time of death did not appear to increase over the years of the study, which was 77 years.

Furthermore, Table 2 describes the subgroups of causes of death. The ten most common causes of death were acute myocardial infarction (53.0%), heart failure (18.0% in total—11.7% as a frank heart failure and 6.3% as a heart failure complicated by the relevant concomitant clinical condition), isolated cardiac arrest (6.6%), sepsis (4.4%), arrhythmia (3.0%), other non-cardiac condition (2.5%), AMI/non-HF complication (2.4%), aortic pathology (2.2%), and infective endocarditis (1.8%). Compared to older patients (age 65 years or greater), younger patients were more likely to die from isolated cardiac arrest (15.7% vs. 4.3%, *p* < 0.001) and other cardiac reasons (3.0% vs. 0.4%, *p* < 0.001). Older patients died more frequently from aortic stenosis (1.6% vs. 0.9%, *p* = 0.39), sepsis (4.6% vs. 3.8%, *p* = 0.62), and heart failure with complications (6.9% vs. 3.8%, *p* = 0.083). Men had a greater proportion of death from sepsis (5.4% vs. 2.8%, *p* = 0.038), heart failure (13.1% vs. 9.6%, *p* = 0.077), and malignancy (1.5% vs. 0.5%, *p* = 0.10) compared to women, but women had proportionately more deaths from aortic stenosis (2.6% vs. 0.8%, *p* = 0.018) and pulmonary embolus (1.6% vs. 0.3%, *p* = 0.012).

**Figure 2:**
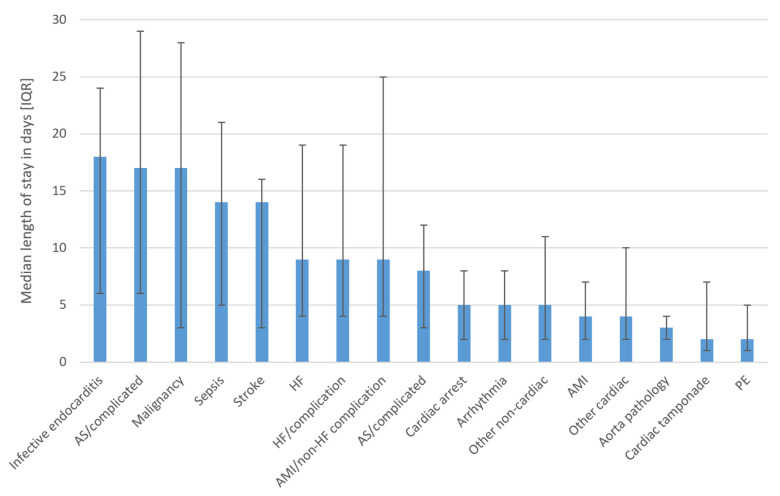
Length of hospital stay in days according to the cause of death. **Abbreviations: AMI**—acute myocardial infarction, **AS**—aortic stenosis; **HF**—heart failure; **PE**—pulmonary embolism.

The median length of stay was greatest where death was associated with infective endocarditis, aortic stenosis with complication, malignancy, sepsis, and stroke (Figure 2). Patients who died from AMI, other cardiac causes, aorta pathology, and cardiac tamponade had the lowest median hospital stay.

**Figure 3:**
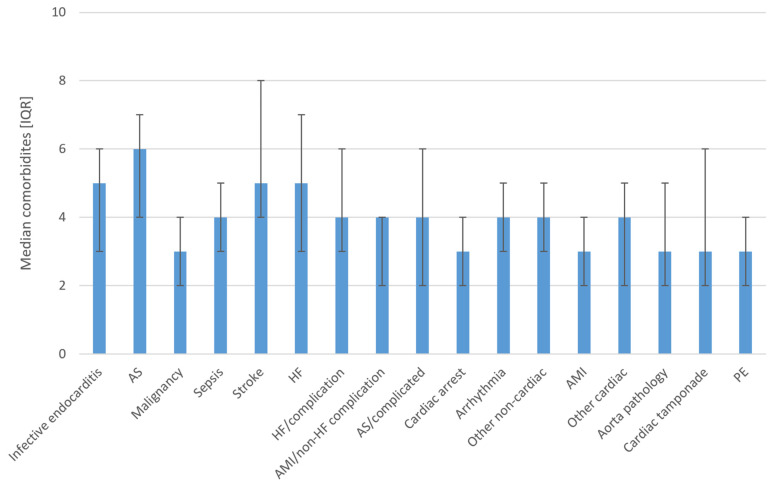
Mean number of comorbidities according to the cause of death. **Abbreviations: AMI**—acute myocardial infarction, **AS**—aortic stenosis; **HF**—heart failure; **PE**—pulmonary embolism.

The number of comorbidities according to cause of death is shown in Figure 3. The median number of comorbidities was greatest for the conditions of aortic stenosis, infective endocarditis, stroke, and heart failure.

The more specific causes of death in subgroups for some of the large cause-of-death clusters are shown in Table 3.

For patients with AMI and non-heart failure complications, the causes of death included valvular heart disease, arrhythmia, bleeding, peripheral vascular disease, bowel complication, sepsis, and other organ dysfunction. More detailed causes of death in heart failure with complications/defined cardiomyopathy included valvular heart disease, defined cardiomyopathy, such as dilated cardiomyopathy or hypertrophic cardiomyopathy, adult congenital heart disease, thrombosis, bleeding, and complete heart block. Aortic pathology was a composite of acute aortic syndrome from dissection, abdominal aortic rupture, thoracic aortic infection, and thoracic aortic occlusion.

**Figure 4:**
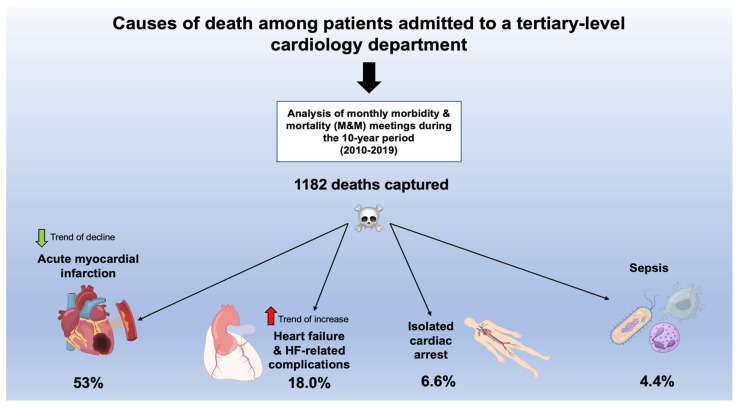
Central figure—A graph summarizing causes of death with the tertiary cardiology department and accompanying trends over the 10-year timespan.

Figure 4 presents the summarized causes of death among patients admitted to a tertiary-level cardiology department.

## 4. Discussion

At our tertiary center, there were 1182 deaths registered over a 10-year period, which is equivalent to an annual average of 118 deaths per year. These deaths were mainly due to AMI and, to a lesser extent, HF and isolated cardiac arrest. In terms of trends, we observed a significant growth in the proportion of deaths from HF and a decline in the proportion due to AMI, with more deaths related to cardiac disease than non-cardiac disease in recent years. An uncommon cause of death was the pathology of the aorta, but there was a notable population who had died from non-cardiac causes, such as sepsis and malignancy (approximately 1 in 10 patients). Moreover, the causes of death varied according to age group, as older patients had a greater proportion of deaths due to aortic stenosis, sepsis, and HF, whereas younger patients were more likely to die of isolated cardiac arrest. The causes of death among younger patients that manifested with an increased proportion of cardiac arrest at presentation might also be partially explained by the sudden cardiac death, as these patients might have had various genetic and hereditary predilections, such as hypertrophic obstructive cardiomyopathy, arrhythmogenic cardiomyopathy, or channelopathies [12,13]. In addition, the length of hospital stay tends to be higher among patients who died of causes such as infective endocarditis, aortic stenosis with complications, malignancy, sepsis, and stroke. These findings suggest that cardiology services should accept that a substantial portion of the mortality will be attributed to AMI, HF, and isolated cardiac arrest, but healthcare professionals should also be prepared to diagnose and manage patients with rare cardiac pathologies and non-cardiac diseases.

Some interesting remarks can be made regarding the trends in mortality observed in our study. It is a well-known fact that mortality due to AMI has decreased significantly worldwide over the last century [12,13,14]. This decline in MI-related deaths is largely attributed to better care of patients with AMI, which has culminated in the contemporary practice of 24/7 primary PCI service and the routine use of potent antithrombotic and secondary prevention medications. The consequences of improved survival among patients with AMI may result in a growing population of patients with ischemic cardiomyopathy who may, later on, present with HF. This is consistent with the epidemiological findings regarding HF mortality, where there has been a growth in the proportion of patients who are dying from HF in the past few decades [15,16]. Although it cannot be exactly clear why HF deaths increased, we can speculate a few reasons. The population where the study took place has become more elderly and comorbid because of good community and hospital care, and many patients are surviving longer and developing HF. There is a primary PCI service that runs 24 h for 7 days a week, which has resulted in good survival rates AMI, but this has resulted in a further increase in the population with HF. HF carries a significantly increased risk of mortality, and a growing population of HF patients will translate into more deaths. Moreover, the area where the study took place has good out-of-hospital emergency services and many nearby hospitals that can stabilize peri-arrest and cardiac-arrest patients before they arrive at the hospital. The impact of this may be that the patients would have died in the community in the absence of such services, but the fact that they survived meant that they would develop or survive with HF, and some of these patients might have survived the acute event but later died in the hospital. While HF care has improved for patients with access to medical treatment, device therapy, and advanced therapy where indicated, the rising population with HF is such that there are some who progress to the stage where no further treatments may be offered, such as those who are frail or comorbid or have dementia, receiving symptomatic supportive care. These high-risk patients with HF are at high risk of death when they are admitted to the hospital, which contributes to the rise in deaths across observed years.

Our study also provides insight into complications and less common but important contributors to death. Pathologies of the aorta, including aortic dissection, thoracic or abdominal aorta rupture, aortic occlusion, and infections of the aneurysms, are contributors to death, which may have had multidisciplinary care with cardiac surgeons. Of importance, it has been recently recognized that deaths from aortic dissection now outnumber deaths from road traffic collisions in the UK [17]. The major single arrhythmia event accounting for death was complete heart blockage, and many of these patients died despite receiving a pacemaker device because of coexisting significant comorbidities and/or frailty. It may well be that cardiac conduction disease is part of an end-stage of other comorbidities and general frailty and, thus, might not have been correctable by pacemaker implantation alone.

We observed that gastrointestinal complications are not uncommon contributors to death among patients admitted to the cardiology department. These pathologies include gastrointestinal bleeding, bowel obstruction, bowel perforation, ischemic colitis, ischemic hepatitis, acute cholangitis, and biliary sepsis. In addition, renal dysfunction is common in AMI and HF patients because of the combination of kidney hypoperfusion and the use of diuretics [18,19]. Infections remain common contributors to death, the most frequent being a chest infection. It was reported that sepsis is the most prevalent cause of death from all hospital admissions in a six-center study in the United States [20]. Furthermore, factors such as advanced frailty, comorbidity, and advanced age seem to be predominant characteristics of hospitalized patients treated for infection, whether they had sepsis or not [21]. Rare causes of death in our sample included chronic multisystem disorders, such as sarcoidosis and amyloidosis, with one patient dying of constrictive pericarditis and one from VT storm. There were also a few cases of patients dying from iatrogenic complications, including infected shunts or implanted devices and cardiac perforation due to pacing lead insertion. Another heterogeneous population was adult patients with congenital heart disease, as there were cases with Eisenmenger syndrome and Tetralogy of Fallot. From an educational perspective, it is important that less-experienced clinicians are aware of how to manage these rare but important pathologies that occur in clinical practice.

We observed differences in the proportion of deaths by age group. Younger patients with underlying heart disease may present for the first time with isolated cardiac arrest. We accept that there may be an age-related selection bias for admissions to a specialist cardiology ward. Older patients who present with cardiac arrest usually have an underlying coronary artery disease, whereas younger patients tend to have structural heart disease and/or arrhythmogenic disorders [22,23,24,25]. Out of these young patients, there was one cardiac arrest event that resulted in death in whom arrhythmogenic right ventricular cardiomyopathy was identified. Similarly, another young patient died after having an isolated cardiac arrest, but it was noted on their records that they had asymptomatic long QT syndrome. There were also two young patients who died from viral dilated cardiomyopathy while waiting for a heart transplant and viral myocarditis that was initially treated as an acute coronary syndrome. On the other hand, older patients had a greater proportion of deaths from aortic stenosis, and some of these patients had undergone transcatheter aortic valve implantation. Furthermore, elderly patients have a significantly greater comorbidity burden and are prone to infections because of frailty [26,27].

This study has some limitations. One of the limits of our analysis is the designation of the cause of death. Firstly, the precise cause of death was not always investigated or confirmed by the autopsy. This was the case of isolated cardiac arrest as it is typically secondary to a pathology, whether it was by a hypoxic, thrombotic, or metabolic mechanism. Secondly, there were also cases where multiple pathologies and comorbidities complicated the exact adjudication of the cause of death. We used a ranking system such that where the history was consistent with AMI as the initial insult causing hospitalization, the cause of death was considered to be AMI even though later non-cardiac complications, such as renal failure or hospital-acquired infection, might have supervened. However, to prevent the omission of these important complications, we included categories of AMI with non-heart failure complications, HF with complications, and AS with complications. Because heart failure is a natural sequela of AMI, we also only considered non-HF-related complications. Also, there were patients who had a life-limiting illness, such as end-stage chronic obstructive pulmonary disease or lung malignancy, who presented with chest pain and cardiac pathology. While end-stage chronic lung disease and malignancy contribute to death, we classified the cause of death as related to acute cardiac pathology unless the history indicated that the non-cardiac problem was the primary cause. In addition, our evaluation was limited to data about the deaths that occurred among inpatients in the department, and we did not know the number of patients who were admitted to hospital over the duration of the evaluation. Finally, the quality of data was limited by the quality of the description from the PowerPoint presentations prepared by the cardiology registrars, and there was a degree of missing data. In some cases, we were able to input missing data by reviewing the electronic medical records. Classification of the cause of death is a complex task, but measures were undertaken to limit potential bias. However, we believe that our findings provide a unique insight into causes of death among patients admitted to the cardiology department at the tertiary medical center derived from morbidity and mortality meetings, as such analysis is unavailable in the literature.

Our findings bear implications for clinical practice. The identification of rare causes of death and discussing their management may help improve future practice for less-experienced clinicians. There is also an educational aspect of M&M meetings, as they provide a learning experience for all physicians involved in patient care, especially younger ones. Also, there are pathologies that benefit from multidisciplinary input; for example, cardiothoracic surgeons can share care for patients with aortic pathology. Furthermore, clinicians in the cardiology department reviewing deteriorating patients need to be aware of potential gastrointestinal complications, as prompt detection and referral for gastroenterology or general surgical input may improve outcomes. There are also training implications as cardiology heads toward more subspecialist training; it remains important that cardiologists are grounded in general medicine and are able to manage general medical emergencies, such as gastrointestinal bleeding and sepsis.

## 5. Conclusions

Taken together, most of the deaths at our cardiology departments were due to AMI, HF, and isolated cardiac arrests, while the proportion of causes of deaths varied depending on the age group, where younger patients had a significantly greater proportion of deaths from isolated cardiac arrest. These findings suggest that tertiary cardiology services should be prepared not only to manage acutely ill patients with AMI, HF, and isolated cardiac arrests but also to investigate and manage patients with less common cardiac pathologies and non-cardiac diseases.

## Figures and Tables

**Table 1:** Trends in causes of death across the 10-year period according to data from morbidity and mortality meetings (percentages (%) and absolute number of deaths)).

CAUSE OF DEATH	2010 (%)	2011 (%)	2012 (%)	2013 (%)	2014 (%)	2015 (%)	2016 (%)	2017 (%)	2018 (%)	2019 (%)
Acute myocardial infarction	61.9	69.5	59.8	61.8	52.3	50.0	58.3	51.0	52.2	46.7
Heart failure	11.1	15.3	13.7	15.1	21.1	23.3	12.5	19.5	17.9	25.9
Aortic stenosis	1.6	1.7	2.6	2.6	0.0	3.3	0.0	4.7	2.2	1.5
Isolated cardiac arrest	1.6	5.1	6.0	6.6	10.1	6.7	7.6	6.7	6.7	5.9
Infective endocarditis	1.6	0.0	0.9	2.0	1.8	2.5	1.4	0.7	4.5	1.5
Arrhythmia	0.0	3.4	3.4	3.9	5.5	0.8	2.1	3.4	3.7	3.0
Cardiac tamponade	0.0	0.0	0.9	0.0	0.0	1.7	0.7	1.3	0.7	1.5
Other cardiac causes	0.0	0.0	0.0	0.0	0.9	0.8	2.1	0.0	2.2	2.2
**Total cardiac (%)**	**77.8**	**94.9**	**87.2**	**92.1**	**91.7**	**89.2**	**84.7**	**87.2**	**90.3**	**88.1**
Pathologies of aorta	6.3	0.0	0.0	2.0	2.8	3.3	3.5	0.7	0.0	4.4
Sepsis	4.8	3.4	7.7	2.0	0.9	4.2	4.9	4.7	6.7	4.4
Pulmonary embolism	3.2	1.7	0.0	0.0	0.9	1.7	1.4	0.7	0.0	0.0
Malignancy	1.6	0.0	1.7	0.0	1.8	0.0	3.5	0.0	2.2	0.0
Stroke	1.6	0.0	0.9	0.0	0.0	0.0	0.0	2.7	0.7	0.0
Other non-cardiac	4.8	0.0	2.6	3.9	1.8	1.7	2.1	4.0	0.0	3.0
**Total non-cardiac (%)**	**22.2**	**5.1**	**12.8**	**7.9**	**8.3**	**10.8**	**15.3**	**12.8**	**9.7**	**11.9**
**Absolute number of deaths**	**63**	**59**	**117**	**152**	**109**	**120**	**144**	**149**	**134**	**135**

**Table 2:** Overview of causes of death according to age and sex.

MAIN GROUP	SUBGROUP	Total (n = 1182)	%	Age < 65 (n = 236)	%	Age ≥ 65 (n = 946)	%	*p*-Value	Men (n = 717)	%	Women (n = 426)	%	*p*-Value
**Acute myocardial infarction** **(N = 654)**	AMI	626	53.0	118	50.0	508	53.7	0.31	367	51.2	232	54.5	0.28
	AMI/non-HF complication	28	2.4	4	1.7	24	2.5	0.45	19	2.6	7	1.6	0.27
**Heart failure (N = 212)**	Heart failure (HF)	138	11.7	24	10.2	114	12.1	0.42	94	13.1	41	9.6	0.08
	HF/complication	74	6.3	9	3.8	65	6.9	0.083	42	5.9	31	7.3	0.34
**Isolated cardiac arrest** **(N = 78)**	Cardiac arrest	78	6.6	37	15.7	41	4.3	<0.001	51	7.1	26	6.1	0.51
**Other cardiac causes** **(N = 102)**	Aortic stenosis (AS)	17	1.4	2	0.9	15	1.6	0.39	6	0.8	11	2.6	0.02
	AS & complication	8	0.7	0	0	8	0.9	0.16	5	0.7	3	0.7	0.99
	Infective endocarditis	21	1.8	3	1.3	18	1.9	0.51	17	2.4	4	0.9	0.08
	Arrhythmia	36	3.0	3	1.3	33	3.5	0.076	18	2.5	16	3.8	0.23
	Cardiac tamponade	9	0.8	3	1.3	6	0.6	0.31	7	1.0	2	0.48	0.35
	Other cardiac cause	11	0.9	7	3.0	4	0.4	<0.001	8	1.1	3	0.7	0.49
**Other non-cardiac causes** **(N = 136)**	Pathology of aorta	26	2.2	4	1.7	22	2.3	0.56	14	2.0	11	2.6	0.48
	Sepsis	52	4.4	9	3.8	43	4.6	0.62	39	5.4	12	2.8	0.04
	Pulmonary embolism	9	0.8	4	1.7	5	0.5	0.065	2	0.3	7	1.6	0.01
	Malignancy	13	1.1	5	2.1	8	0.9	0.093	11	1.5	2	0.5	0.10
	Stroke	7	0.6	1	0.4	6	0.6	0.71	2	0.3	4	0.9	0.14
	Other non-cardiac	29	2.5	3	1.3	26	2.8	0.19	15	2.1	14	3.3	0.21

**Table 3:** Specific registered complications across relevant major cause of death subgroups.

Acute Myocardial Infarction		Aortic Stenosis		Pathologies of Aorta		Arrhythmia	
Valvular heart disease Arrhythmia Bleeding Peripheral vascular disease Bowel complication Sepsis Other organ dysfunction	4 1 3 1 6 6 7	Bowel complication Arrhythmia Sepsis	2 2 4	AAA rupture Thoracic aorta occlusion Thoracic acute aorta syndrome Thoracic aorta infection	3 1 19 3	Tachycardia Bradycardia	13 23
**Total**	**28**	**Total**	**8**	**Total**	**26**	**Total**	**36**
**Heart failure**		**Sepsis**		**Other cardiac causes**		**Other non-cardiac causes**	
Valvular heart disease Defined cardiomyopathy or adult congenital heart disease Sepsis Other organ dysfunction Thrombosis/bleeding/hematological problem Complete heart block	20 5 31 11 5 2	Gastrointestinal disease Skin and limb Pneumonia Device-related Unclear source Urosepsis	3 3 19 1 23 3	Adult congenital heart disease Constriction Device-related Other	2 1 3 5	Other organ dysfunction Peripheral vascular disease Infection Abdomino-pelvic bleeding or ischemia Cerebral bleed Vascular disease Other	6 2 3 5 8 1 4
**Total**	**74**	**Total**	**52**	**Total**	**26**	**Total**	**36**

## Data Availability

Data are available upon reasonable request.
